# Multi-parameter comparison of a standardized mixed meal tolerance test in healthy and type 2 diabetic subjects: the PhenFlex challenge

**DOI:** 10.1186/s12263-017-0570-6

**Published:** 2017-08-29

**Authors:** Suzan Wopereis, Johanna H. M. Stroeve, Annette Stafleu, Gertruud C. M. Bakker, Jacobus Burggraaf, Marjan J. van Erk, Linette Pellis, Ruud Boessen, Alwine A. F. Kardinaal, Ben van Ommen

**Affiliations:** 1 TNO, Netherlands Institute for Applied Scientific Research, Zeist, The Netherlands; 2 Centre for Human Drug Research (CHDR), Leiden, The Netherlands

**Keywords:** Nutrition, Phenotypic flexibility, Biomarkers, Nutritional challenge test, Metabolic health, Type 2 diabetes

## Abstract

**Background:**

A key feature of metabolic health is the ability to adapt upon dietary perturbations. Recently, it was shown that metabolic challenge tests in combination with the new generation biomarkers allow the simultaneous quantification of major metabolic health processes. Currently, applied challenge tests are largely non-standardized. A systematic review defined an optimal nutritional challenge test, the “PhenFlex test” (PFT). This study aimed to prove that PFT modulates all relevant processes governing metabolic health thereby allowing to distinguish subjects with different metabolic health status. Therefore, 20 healthy and 20 type 2 diabetic (T2D) male subjects were challenged both by PFT and oral glucose tolerance test (OGTT). During the 8-h response time course, 132 parameters were quantified that report on 26 metabolic processes distributed over 7 organs (gut, liver, adipose, pancreas, vasculature, muscle, kidney) and systemic stress.

**Results:**

In healthy subjects, 110 of the 132 parameters showed a time course response. Patients with T2D showed 18 parameters to be significantly different after overnight fasting compared to healthy subjects, while 58 parameters were different in the post-challenge time course after the PFT. This demonstrates the added value of PFT in distinguishing subjects with different health status. The OGTT and PFT response was highly comparable for glucose metabolism as identical amounts of glucose were present in both challenge tests. Yet the PFT reports on additional processes, including vasculature, systemic stress, and metabolic flexibility.

**Conclusion:**

The PFT enables the quantification of all relevant metabolic processes involved in maintaining or regaining homeostasis of metabolic health. Studying both healthy subjects and subjects with impaired metabolic health showed that the PFT revealed new processes laying underneath health. This study provides the first evidence towards adopting the PFT as gold standard in nutrition research.

**Electronic supplementary material:**

The online version of this article (10.1186/s12263-017-0570-6) contains supplementary material, which is available to authorized users.

## Background

A key feature of health is the ability to adapt upon a large variety of perturbations [1, 2]. In the context of metabolic health, these perturbations primarily come from our diet. The quantification of the multitude of responses upon a metabolic meal tolerance test (carbohydrate, lipid, and protein) reveals detailed insight in mechanisms and organs involved in maintaining metabolic homeostasis [3] and is specified as “phenotypic flexibility” [4].

The use of emerging technologies like metabolic profiling and targeted proteomics in metabolic tolerance tests has allowed the development of a new generation of biomarkers [5] by the simultaneous quantification of multiple processes [3, 6–11]. The response to challenges may be used to derive biomarkers for maintenance of physiological function and ultimately as indicator for prevention of (metabolic) diseases. There is a strongly increasing interest in this type of biomarker in nutrition and health studies, demonstrating their use in uncovering early alterations in metabolism preceeding chronic diseases [9, 11–14]. Recently, it was shown that quantification of challenge response significantly contributes to demonstrating health effects of food and nutrition in dietary intervention studies [6, 7, 14–18]. A standardized optimal nutritional challenge test was defined after performance of a systematic literature review [3]. In this literature review, modulation of 35 different physiological processes were being evaluated. This covered 61 studies that applied various metabolic challenge tests to quantify health and nutritional effects, with the objective to develop a standard in nutrition and health research [3]. This challenge test was named the “PhenFlex test” (PFT).

The present study aimed to assess the ability of PFT to quantify flexibility of a multitude of processes, both in healthy and metabolically impaired subjects and therefore evaluated PFT response in 20 male volunteers with type 2 diabetes mellitus (T2D) to 20 healthy male volunteers. Processes included gut hormone production, lipolysis and adipokine production, (re)absorption, urea cycle, endothelial integrity, protein/amino acid metabolism, muscle tissue injury control, core metabolism, lipoprotein production, hepatic tissue injury control, α- and β cell function, systemic insulin sensitivity, oxidative stress, and switch between carbohydrate and lipid oxidation. Our hypothesis was that the biomarker PFT response would identify the involvement of multiple processes in T2D which are not shown at fasting nor in healthy subjects. Finally, we compared PFT to an OGTT as a benchmark for glucose metabolism to investigate if similar responses would occur upon both challenges.

## Methods

### Subjects

The study was conducted at the Centre of Human Drug Research (CHDR) in Leiden, The Netherlands. Study participants were recruited from the CHDR volunteer database and via study specific advertisements in local media and internet. The trial was retrospectively registered on 25 April 2017 with ID: ISRCTN27707180.

The study population consisted of two groups of 20 male participants aged 30–70 years. The first group consisted of 20 healthy male volunteers with a body mass index (BMI) between 20.0–25.0 kg/m^2^. The second group consisted of 20 male T2D subjects with a documented history of diabetes mellitus and use of prescription oral glucose-lowering drug(s). The T2D had a BMI of 25.1–34.9 kg/m^2^ and stopped antidiabetic medication 1 week prior to the first study day. All subjects reported to have regular Dutch eating habits. Excluded were subjects with uncontrolled hypertension (systolic blood pressure ≥ 150 mmHg and/or diastolic blood pressure of ≥ 95 mmHg), having a history of medical surgical events other than T2D that might significantly affect the study outcome, use of medication that might interfere with parameters to be measured with one of the challenge tests, smokers, active in sports for more than 6 h a week, reported unexplained weight loss or gain > 4 kg in the month prior to the pre-study screening, reported slimming or medically prescribed diet, or a reported food allergy or sensitivity. For the diabetic subjects, additional exclusion criteria were use of insulin and a fasting glucose of < 7 mM after stopping oral anti diabetic treatment for 1 week. The study was performed in compliance with good clinical practice.

### Study design

**Fig. 1:**
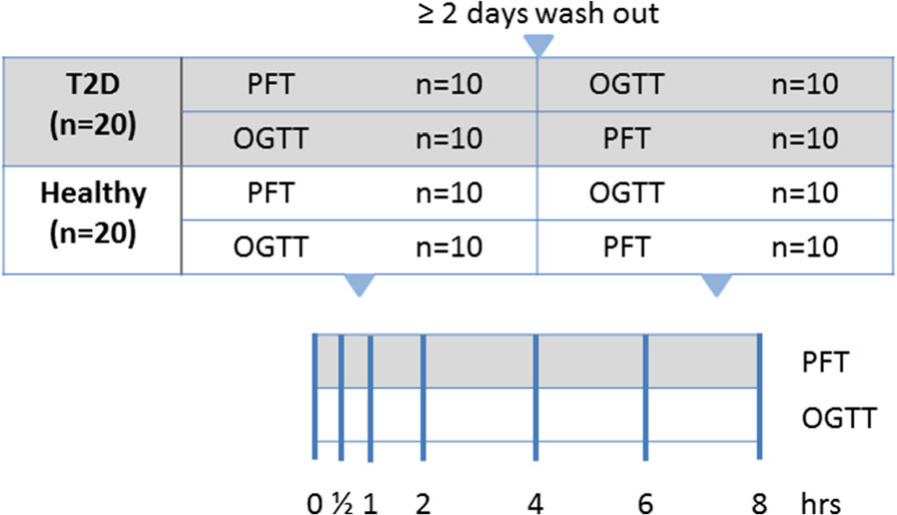
Overview of study design and time points at which blood samples were collected in response to challenge tests for biomarker analysis. Twenty healthy male volunteers and 20 T2D subjects were given the PhenFlex test (PFT) drink or a glucose drink (OGTT) in randomized order on two different study days. The wash-out period between the two challenge tests was at least 2 days. On study days, blood samples were taken at *t* = 0 (≥ 10 h of fasting) and six time points (*t* = 0.5, *t* = 1, *t* = 2, *t* = 4, *t* = 6, and *t* = 8 h) after consumption of the challenge drinks for analysis of a total of 132 different metabolic markers

The study was a explorative randomized cross-over study in two groups of 20 healthy male volunteers and 20 male T2D subjects (Fig. 1). The sample size was not determined by statistical power analysis but was based on the results of previous studies with a similar study design [6, 19–21]. To minimize the risk of bias and enhance the validity of statistical comparisons, we choose to only include male subjects in this explorative study. Both groups were given the PhenFlex test (PFT) drink or a glucose drink (OGTT) in the morning after an overnight fast (≥ 10 h) in randomized order on two different study days. The wash-out period between the two challenge tests was at least 2 days. The wash-out period was sufficient, which was statistically checked by comparing baseline biomarker concentrations within subjects. On study days before the first blood draw, a cannula was placed and blood samples were taken at *t* = 0 (fasting) and six time points (*t* = 0.5, *t* = 1, *t* = 2, *t* = 4, *t* = 6, and *t* = 8 h) after consumption of the challenge drinks. Subjects were not allowed to eat or drink until the last blood sampling, except from drinking water. In addition to the blood sampling, ventilated hood measurements were performed in subgroups of subjects. A subgroup of ten healthy and ten T2D underwent an indirect calorimetry measurement to assess the metabolic flexibility (QUARK).

Subjects were instructed to eat the same meal on the evening before each study day and to maintain their habitual life style and diet from the screening until the end of the study period, with the following restriction: no unusual exercise/physical activity, no intake of food supplements, NSAIDs, betablockers, statin, or paracetamol. Use of ACE-inhibitors and statins was allowed for the T2D during the study.

### Challenge drinks

The PhenFlex test used a drink (the “PhenFlex drink”) of 400 mL, which consisted of a mixture of 12.40% (*w*/*w*) palm olein, 17.25% (*w*/*w*) dextrose, 4.13% (*w*/*w*) Protifar (Nutricia), 0.10% (*w*/*w*) (vanilla flavor), 0.12% (*w*/*w*) trisodiumcitrate, 0.08% (*w*/*w*) sodiumhydroxide, and 66.12% (*w*/*w*) water. This resulted in a drink of 3950 kJ/950 kcal with a macronutrient composition of 60 g fat (of which 39% saturated fatty acids, 47% mono unsaturated fatty acids, 14% poly unsaturated fatty acids), 75 g glucose, and 20 g protein. The PhenFlex drink was food-grade produced using a production protocol with HACCP principles in the NIZO food research processing centre. The OGTT was a standard oral glucose tolerance test consisting of 75 g glucose in 300 mL water prepared by the LUMC hospital pharmacy. The drinks were stored in a refrigerator with restricted access at 2–8 °C until consumption. Both challenge drinks had to be consumed within 5 min.

### Metabolic parameters

Blood samples were collected in tubes containing clot activator for serum or in ice-chilled tubes containing Li-heparin or K_2_EDTA as anticoagulant for plasma and whole blood. DPP-IV inhibitor was added to K_2_EDTA tubes for glucose-dependent insulinotropic polypeptide (GIP) analyses and approtinin to tubes for glucose-related parameters. After centrifugation (for 15 min at approximately 2000 g at approximately 4 °C within 30 min after collection), plasma and serum samples were stored at ≤ − 20 °C for clinical chemistry and ≤ − 70 °C for all other parameters. The following parameters were measured using routine methodology in all serum samples: total cholesterol, HDL cholesterol, LDL cholesterol, triglycerides, free fatty acids (FFA), glucose, gamma-glutamyltransferase (GGT), alanine-aminotransferase (ALAT), aspartate-aminotransferase (ASAT), alkaline phosphatase (ALP), albumin, and creatinine. Plasma samples were analyzed for C-reactive protein (CRP), serum amyloid A (SAA), secreted intercellular adhesion molecule-1 (sICAM-1), secreted vascular adhesion molecule-1 (sVCAM-1) using a 4-plex Meso Scale Discovery (MSD, Rockville, Maryland, USA). Glucose-related parameters consisting of glucagon, glucagon-like peptide-1 (GLP-1), leptin, and insulin were measured in plasma using a different 4-plex MSD plate (MSD, Rockville, MD, USA). Enzyme-linked immunosorbent assays (96-wells) were used to measure adiponectin, GIP, C-peptide, and glutathione ratio. Metabolic profiling was used for assessment of endogenous plasma metabolites by gas chromatography–mass spectrometry (GCMS) technology. In addition, fasting glycated hemoglobin A1c (HbA1c) (in whole blood) and fructosamine (in serum) were measured. All parameters were analyzed by TNO Triskelion BV.

### Indirect calorimetry

Substrate oxidation was measured with the ventilated hood method (QUARK RMR, version 9.1, Cosmed, Rome, Italy) according to the manufacturer’s instructions. This indirect calorimetry is based on measurement of gas exchange, to reflect energy expenditure and nutrient metabolism [22]. The head of the patient is covered with a transparent plastic canopy hood, connected to a blower, generating a constant flow through the hood. Inhaled air enters from the surrounding environment (room air) and the exhaled O_2_ and CO_2_ content is measured for calculation of O_2_ consumption and CO_2_ production. The respiratory quotient (RQ) was assessed as the ratio of CO_2_ exhaled to the amount of oxygen consumed by the individual (RQ = VCO_2_/VO_2_). Before consumption of the challenge drink, a measurement of 20 min was performed, reflecting the fasting substrate oxidation of the subjects. Data of the first 5 min were discarded. From the subsequent period of 15 min, a 10-min reading was selected which reflected a steady state. After consumption of the challenge drink, substrate oxidation was measured continuously for 3 h. Data reflecting the most stable 10 min from the second half of every 30-min period were used to calculate substrate oxidation at *t* = 30, 60, 90, 120, 150, and 180 min.

### Body composition

Whole-body electrical resistance measurements were performed for assessing body composition using an InBody 720 body composition analyzer (InBody, Seoul, Korea). Bio-impedance was measured via resistance in broadband frequencies of 1 kHz–1 MHz and reactance in mean frequencies. Based on the data of the bio-impedance measurement, body composition was measured indirectly. Fat mass of the body was computed as body weight minus fat-free mass.

### Insulin resistance, insulin secretion, and β cell function

A number of indexes related to insulin resistance, insulin secretion, and β cell function were calculated using previously published methods. The Hepatic Insulin Resistance Index (HIRI) was calculated as fasting insulin (mU/L) × fasting glucose (mg/dL) [23], and Muscle Insulin Sensitivity Index (MISI) was calculated as the rate of decay of plasma glucose concentration from its peak value to its nadir during the OGTT divided by the mean plasma insulin concentration [24]. Adipocyte insulin resistance was calculated as fasting plasma insulin (mU/L) × fasting plasma non-esterified fatty acids (NEFA) (mmol/L) [25].

Insulin sensitivity was calculated by two methods: the Matsuda Index (ISI) = 10,000/√ [(fasting insulin (mU/L) × fasting glucose (mg/dL)) × (mean OGTT insulin (mU/L) × (mean OGTT glucose (mg/dL) [23] and the Homeostasis Model Assessment of Insulin Resistance (HOMA-IR) = fasting insulin (mU/L) × fasting glucose (mmol/L)/22.5 [26].

β cell function was determined by two methods: first through calculation of the Disposition Index = ISI × (AUC_0–30_ Ins/AUC_0–30_ Glc), where AUC_0–30_ is the area under the curve between baseline and 30 min of the OGTT for insulin (mU/L) and glucose (mg/dL) measurements, respectively, calculated by the trapezoidal method [27]. Secondly, by the homeostasis model assessment of beta cell function that estimates steady-state beta cell function (%B) (HOMA-B) = (20 × fasting insulin mU/L)/(fasting glucose (mmol/L) −3.5) [26]. Insulin secretion was measured by the insulinogenic index (IGI): IGI = [30 min insulin − fasting insulin (mU/L)]/[30 min glucose − fasting glucose (mmol/L)] [28].

### Statistics

Statistical analyses were performed using the Statistical Analysis System software package version 8.2 (SAS Institute, Cary, NC, USA). Means and standard deviations were calculated for blood parameters and parameters regarding the substrate oxidation. Several area under the curve (AUC) parameters were calculated using the first measurement (*t* = 0) as reference (M1 method) exactly as described by Pellis et al. [7]. ANOVA was applied with the interaction subject group × time as main effect. Time curves for healthy subjects were compared to time curves for T2D. In case of a significant interaction, the response in time upon the PFT or OGTT was investigated for both groups (healthy and T2D). In case of no significant interaction between healthy/T2D and time, the response in time upon the PFT/OGTT is equal for both groups. Main time and group effects were investigated to study overall time and group effects. The AUC measurements for healthy subjects were compared to the AUC measurements for T2D.

For all ANOVAs, plots of residuals versus the corresponding fitted values were inspected. If these plots revealed a residual variation that increases with the fitted value, the data were transformed by taking their natural logarithm. The transformation was performed on the original data set. If the absolute value of a residual exceeded three times the residual standard deviation, the corresponding data point was flagged as an outlier and was removed from the data set.

The null hypotheses (no effect) were rejected at the 0.05 level of probability (α = 0.05). *P* values (interactions between healthy/T2D and time, AUCtotal and *T* = 0) were collected into one table and corrected for testing multiple parameters by applying the Benjamini–Hochberg procedure for controlling the false discovery rate (FDR). The analysis of the other AUC characteristics (AUC+, AUC−, Tmin, Tmax, Cmin, Cmax) was used as additional support for the findings on AUCtotal. Biomarkers that had a significant interaction effect (time × group response) or a significant AUC effect had a differential response to the PFT or showed curve shape differences were referred to as the challenge response. Biomarkers that had a differential group effect had differential offset concentrations but the same shape of the challenge response curve over the time course was referred to as postprandial concentrations or postprandial levels response.

### Hierarchical clustering

The same method was used as described in Pellis et al. to create groups of parameters exhibiting a similar response to the PFT [7]. Hierarchical clustering was performed on the time profiles of all parameters with a significant time effect. For the hierarchical clustering, Pearson’s correlation was used as the distance measure, and complete linkage was used to define the distance between clusters. The number of five clusters was chosen manually taking into account the difference in response and the number of plasma parameters. We aimed to define clusters clearly distinct in the type of response and containing at least four parameters.

## Results and discussion

### Subjects characteristics

The T2D were older (*p* < 0.0001; average age 58 years) and had higher BMI (*p* < 0.0001; 29 kg/m^2^) compared to the healthy subjects (average age 42 years and BMI 23 kg/m^2^) (Table 1). Fasting glucose levels were higher (*p* < 0.0001) in T2D (average of 10.2 mmol/L) compared to the healthy subjects (average of 5.4 mmol/L).

**Table 1:** Demographic characteristics and fasting laboratory values of subjects at inclusion (mean (SD))

	Healthy (*n* = 20)	T2D (*n* = 20)
Age (years)	42.2 (7.6)	58.3 (9.1)^***^
BMI (kg/m^2^)	23.3 (1.5)	28.7 (2.2)^***^
Glucose (mmol/L)	5.4 (0.5)	10.2 (1.7)^***^
Cholesterol (mmol/L)	5.0 (1.0)	4.6 (1.2)
HDL (mmol/L)	1.3 (0.3)	1.0 (0.2)
LDL (mmol/L)	3.4 (0.8)	3.1 (1.0)
Triglyceride (mmol/L)	1.4 (1.0)	1.9 (0.9)

^*^Significant differences between fasting values healthy subjects and T2D (*p* value < 0.05);

^**^Significant differences between fasting values healthy subjects and T2D (*p* value < 0.01);

^***^Significant differences between fasting values healthy subjects and T2D (*p* value < 0.001)

### PhenFlex test response characterization in healthy subjects

**Fig. 2:**
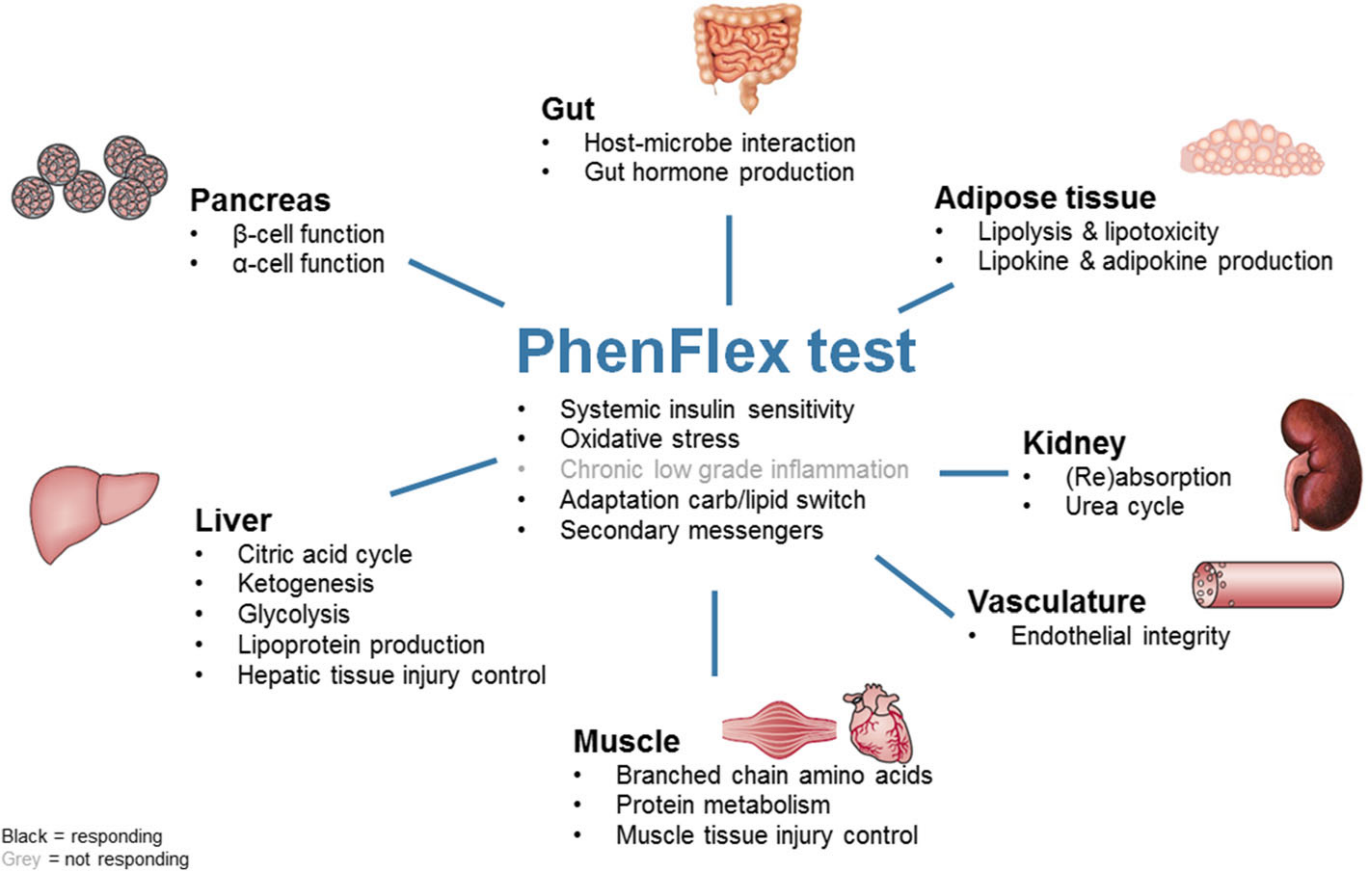
Overview of processes that are modulated by the PhenFlex test based on biomarker response in *n* = 20 healthy male subjects. Black: modulated by PFT; gray: not responding

**Fig. 3:**
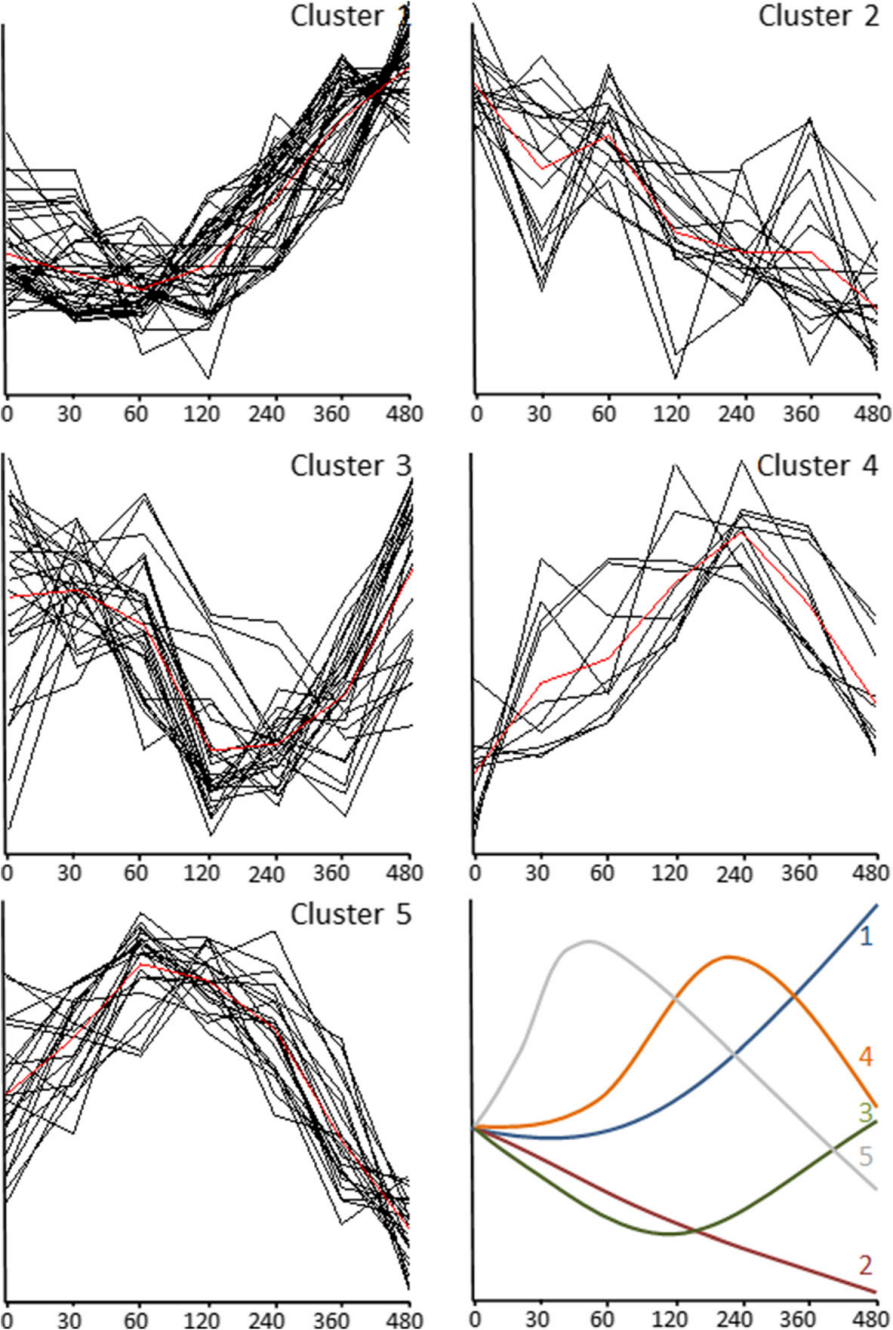
The five different observed time course clusters in response to the PhenFlex test, based on the 110 plasma metabolites and proteins with a significant effect in time. The red line represents the average cluster time profile. The *x*-axes were expressed as time (minutes), the *y*-axes were expressed as relatively scaled concentrations. Parameters from cluster 1 increased during most of the 8-h time course. Parameters from cluster 2 decreased during most of the time course of 8 h. Parameters from clusters 3 decreased upon PFT, with a subsequent recovery phase. Parameters from cluster 4 showed a classical absorption profile. Parameters from cluster 5 reached maximum values around 1 h and minimum concentrations were reached at the final time point (8 h). Finally, the different average cluster curves are summarized in one figure

Statistical analysis revealed that 110 of 132 parameters responded significantly to PFT drink in healthy subjects (Additional file 1: Table S1, Fig. 2). These 110 significantly modulated plasma parameters were grouped into 5 discrete response time course clusters (Fig. 3). Cluster 1 was the biggest cluster represented by 37 out of 110 plasma parameters. These parameters increased during most of the 8 h time course. Cluster 2 represents 8 plasma parameters that mainly decreased during most of the time course of 8 h. Time profile cluster 3 represented the 31 plasma parameters that decreased upon PFT, with a subsequent recovery phase. Cluster 4 represented 8 plasma parameters with a classical absorption profile, reaching maximum concentrations around 4 h after PFT challenge, followed by a continued reduction towards baseline values at the final (8 h) time point. Cluster 5, represented by 26 plasma parameters has a similar profile to cluster 4, with the main difference that parameters in cluster 5 reached maximum values around 1 h and that minimum concentrations were reached at the final time point (8 h), which were below baseline values. The healthy physiological responses of main metabolic processes modulated by PFT are described in more detail below.

#### Gut

##### Gut hormone production

In response to the lipid load, the gut secretes the incretin GIP. GIP shows a temporary increase in response to PFT drink, with maximum concentrations 4 h postprandially (cluster 4), similar as to TG response profile. Also, GLP-1 showed a similar time profile as compared to GIP (cluster 4).

#### Adipose tissue

##### Lipolysis

Plasma concentrations of both NEFA and glycerol increased after a lag time (cluster 1). Insulin, immediately released after PFT intake, suppresses fat mobilization for energy production. In the late (catabolic) phase of the 8-h time course, adipose tissue triglycerides are hydrolyzed by hormone-sensitive lipase for beta-oxidation, evidenced by the observed increased plasma levels of NEFA and glycerol. The various plasma free fatty acids either showed the same time cluster 1 response as NEFA or decreased plasma concentrations with subsequent recovery (cluster 3 response). Subsequently, also the plasma monoglycerides and diglycerides showed increased concentrations after a lag time (cluster 1).

##### Adipokine production

Leptin, the hormone that regulates the amount of fat storage, is secreted when the amount of fat storage has reached a certain threshold. This adipokine showed increased concentrations in the late time frame of PFT response (cluster 1).

#### Kidney

##### (Re)absorption

Measuring serum creatinine is the most commonly used indicator of renal function. Creatinine showed decreased concentrations in response to PFT (cluster 2).

##### Urea cycle

Urea, which is formed in the urea cycle by deamination of amino acids in the liver, is a waste product excreted by the kidney in the urine. Also, urea showed decreased concentrations in response to PFT (cluster 2). Together, these observations suggest that glomerular filtration rate of kidneys increased in response to PFT.

#### Vasculature

##### Endothelial integrity

The plasma total, HDL, and LDL cholesterol concentrations decreased in response to PFT with subsequent recovery (cluster 3). Also, the plasma adhesion markers sICAM and sVCAM showed the same response to PFT as compared to cholesterol parameters. Finally, SAA showed decreased concentrations in response to PFT (cluster 2). Together, this suggests that there was a temporarily reduced vascular response after PFT.

#### Muscle

##### Protein metabolism

Most plasma amino acids showed a classic absorption profile in response to PFT (cluster 5). This is a rapid increase which returns to baseline within 4–5 h and with concentrations below fasting concentration at the final time point (8 h). Some of the amino acids, however, showed decreased concentrations in response to PFT (glycine and tryptophan, cluster 2).

##### Muscle tissue injury control

The amino acid derivatives 3-methylhistidine and 1-methylhistidine together with creatinine that originate from muscle showed linear decreasing concentrations in response to PFT (cluster 2). Together, this suggests that muscle turns into an anabolic state after consumption of PFT.

#### Liver

##### Core metabolism

The *glycolysis* intermediate pyruvate showed a classic absorption profile (cluster 5), similar to glucose and most amino acids. Glycerol-3-phosphate, an intermediate metabolite derived from glycolysis, accumulated in plasma (cluster 1 profile). Plasma lactate showed linear decreased concentrations (cluster 2). This suggests that ATP was mainly aerobically produced and that the process of oxidative phosphorylation may have reached its maximum capacity. The TCA cycle intermediates succinate, malate, and citrate showed temporarily decreased plasma concentrations in response to PFT (cluster 3), whereas alpha-ketoglutarate showed increased concentrations after a lag phase (cluster 1) in response to PFT.

##### Lipoprotein production

Hepatic very low-density lipoprotein (VLDL) production (represented by free cholesterol and sphingomyelins) showed continuously increasing concentrations in response to PFT after a lag phase of about 2 h (cluster 1). Highest levels were reached at the final time point (8 h). After about 4 h maximum TG, plasma concentrations were reached (cluster 4), which were normalized at the final time point (8 h).

##### Hepatic tissue injury control

Similar cluster 1 responses were observed for liver integrity enzymes (ALAT, ASAT, GGT, ALP) and the process of *ketogenesis* (3-hydroxybutanoic acid, acetoacetate, 2-hydroxybutanoic acid).

#### Pancreas

##### β cell function

PFT caused a temporary release of both C-peptide and insulin from the pancreatic beta cells into the circulation with maximum concentrations around 1 h (cluster 5).

##### α cell function

Glucagon also showed a temporary release during PFT but at a later stage with maximum concentrations around 4 h (cluster 4).

#### Systemic stress

##### Systemic insulin sensitivity

PFT drink that contained 75 g of glucose (33 E%) caused a temporary increase in plasma concentrations of both glucose and fructose (cluster 5).

##### Oxidative stress

Several markers related to oxidative stress responded to PFT. The antioxidant uric acid showed temporarily increased concentrations (cluster 5), whereas the antioxidant vitamin E and mannose (ER stress) showed temporary reduced concentrations (cluster 3 response). Finally, erythronic acid, a molecule formed when *N*-acetyl-d-glucosamine is oxidized, showed temporarily increased concentrations in the late phase of the time course (cluster 4 response).

##### Adaptation carbohydrate and lipid switch

The RQ showed a significant increase in response to PFT drink with maximum reached at 2 h. RQ returned to fasting values at 150 min.

### Comparison of healthy vs. T2D subjects at fasting

Levels of several biomarkers were significantly different in T2D as compared to healthy subjects at fasting (Table 2). A total of 18 out of 132 parameters were statistically different in T2D as compared to healthy subjects.

**Table 2:** Fasting levels of markers that were significantly different between 20 healthy male subjects and 20 type 2 diabetic male subjects, related to adipose tissue, vasculature, kidney, muscle, liver, pancreas, and systemic stress

	Parameter	Unit	Healthy	T2D
Adipose tissue	Leptin	ng/mL	2.96 (2.7)	6.53 (4.2)^**^
	Adipose IR index	–	2.80 (0.12)	3.86 (0.12)^***^
Vasculature	sICAM-1	ng/mL	247 (38)	299 (56)^*^
Kidney	Ornithine	RC	0.14 (0.02)	0.18 (0.04)^*^
	1,5-anhydroglucitol	RC	1.10 (0.2)	0.44 (0.2)^***^
Muscle	Leucine	RC	1.16 (0.2)	1.33 (0.2)^*^
	Valine	RC	1.88 (0.3)	2.16 (0.2)^*^
	4-Oxoproline	RC	0.019 (0.006)	0.011 (0.005)^**^
	Glycine	RC	1.09 (0.2)	0.91 (0.2)^*^
	Glutamate	RC	0.28 (0.1)	0.49 (0.2)^**^
Liver	ALAT	U/L	22.3 (9.8)	38.0 (23.8)^***^
	GGT	U/L	21.4 (8.9)	36.7 (16.6)^***^
	2-Hydroxybutanoic acid	RC	0.078 (0.03)	0.13 (0.04)^***^
	Liver IR index	–	902 (565)	3348 (1312)^***^
Pancreas	Insulin	ng/mL	0.31 (0.2)	0.60 (0.2)^***^
	HOMA-B	%	94 (62)	50 (24)^**^
Systemic stress	HbA1c	ng/mL	95.0 (23)	127 (29)^**^
	HOMA-IR	–	2.23 (1.4)	8.27 (3.2)^***^
	Glucose	mmol/L	5.4 (0.5)	10.2 (1.7)^***^
	Fructose	RC	0.034 (0.01)	0.059 (0.02)^***^
	Mannose	RC	0.33 (0.05)	0.58 (0.1)^***^
	CRP	ng/mL	567 (395)	1342 (1028)^*^

Data are presented as mean ± SD

*RC* relative concentrations against internal standard

^*^Significant differences between fasting values healthy subjects and T2D (*p* value < 0.05);

^**^Significant differences between fasting values healthy subjects and T2D (*p* value < 0.01);

^***^Significant differences between fasting values healthy subjects and T2D (*p* value < 0.001)

#### Glucose metabolism

Markers related to insulin and glucose metabolism were significantly different between the T2D and healthy subjects at fasting such as increased glucose, insulin, and HbA1C. All fasting indexes (lower HOMA-B; higher HOMA-IR, HIRI, and AIR) were also different between the two subject groups. Other plasma metabolites related to glucose metabolism showed lower (1,5-anhydroglucitol, glycine) or higher (mannose, fructose, valine, leucine, 2-hydroxybutanoic acid) plasma concentrations in T2D.

#### Lipokine and adipokine production

Leptin concentrations were significantly higher in the T2D patients, suggesting higher adiposity, which was confirmed by a higher BMI in T2D.

#### Liver integrity

Plasma concentrations of ALAT and GGT were found to be significantly elevated as compared to the healthy volunteers at fasting, suggesting reduced liver integrity in T2D.

#### Inflammatory state

Higher levels of CRP and sICAM were found, suggesting an inflammatory state in T2D.

### Comparison of healthy vs. T2D subjects in response to PFT

**Fig. 4:**
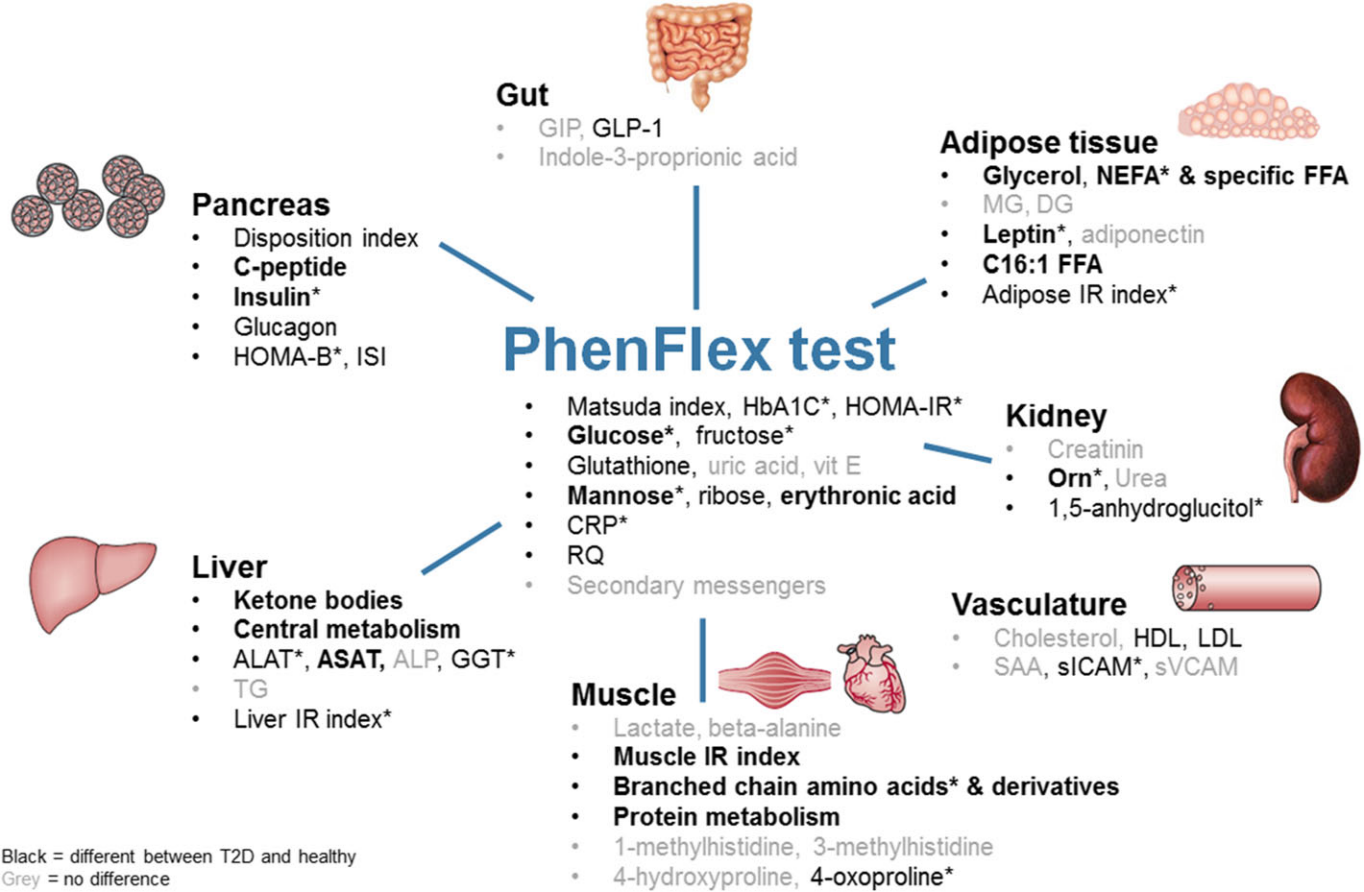
Overview of markers that have a different PhenFlex test response between 20 healthy male and 20 male type 2 diabetic patients. Gray = no significant differences between T2D and healthy subjects; black = significant different postprandial levels between healthy and diabetic subjects; bold black = significantly different responses to PhenFlex challenge between healthy and type 2 diabetics; asterisk = significant different fasting levels

A total of 58 out of 132 parameters showed a statistically different challenge effect in T2D as compared to healthy subjects (Fig. 4). Furthermore, four PFT-based indexes related to insulin sensitivity and β cell functioning (IGI, DI, ISI, and MISI) were also statistically different (Additional file 1: Table S1; Additional file 2: Figures S1 A–F). The responses of the phenotypic processes that were differentially modulated by PFT in T2D as compared to healthy controls are described in more detail below.

#### Glucose metabolism

The insulin and C-peptide PFT response was significantly different in T2D as compared to healthy subjects. A delayed insulin response was found with maximum concentrations at the 2-h time point in T2D, as compared to the 1-h time point in healthy subjects. For C-peptide, maximum concentrations were reached at the 4-h time point in T2D, as compared to the 2-h time point in healthy subjects. DI and IGI indicated a decreased *β cell function,* whereas the *α cell* of the pancreas secreted higher levels of the hormone glucagon in T2D. Subsequently, T2D had a higher glucose PFT response with higher maximum concentrations as well as decreased ISI, indicating reduced *systemic insulin sensitivity* in T2D. The *gut hormone* GLP-1, closely connected to glucose metabolism, showed higher maximum concentrations as well as a faster return to homeostatic levels in T2D as compared to healthy subjects.

#### Adipose tissue

The response to PFT revealed a diminished *lipolysis rate* and *leptin response* in T2D as compared to healthy subjects. A blunted NEFA and glycerol response was observed in T2D. The individual non-essential FFAs showed the same blunted response profiles (C12:0, C14:0, C16:0, C16:1, C17:0, C18:0, C18:1 and C18:2) in T2D, whereas this was not the case for the essential FFA (C20:4 and C22:6). No difference in response was observed for the essential FFA between T2D and healthy male volunteers.

#### Muscle

A distinctly different response to PFT between T2D and healthy subjects was observed for most proteinogenic amino acids. This suggests a different *protein metabolism* between the two groups. Fourteen out of 19 proteinogenic amino acids showed a differential PFT response. T2D showed for all plasma BCAAs and derivatives a higher amplitude in response to PFT as compared to healthy subjects. Similar differences in PFT response profiles were observed for serine, lysine, threonine, glutamate, and tyrosine. Finally, alanine, asparagine, glutamine, cysteine, and phenylalanine also showed a differential PFT response in T2D as compared to healthy that differed from the above described amino acids.

Three metabolites that are intermediates of *energy metabolism* responded differently to PFT between T2D and healthy subjects. These were alpha-ketoglutarate, succinate, and pyruvate. Together, PFT response profiles from these metabolites suggest that T2D have a diminished carbohydrate metabolism shown by higher amplitudes for glycolysis and TCA cycle as compared to healthy subjects.

#### Liver

The ketone bodies 3-hydroxybutanoic acid and acetoacetate both involved in *ketogenesis* showed a reduced response in T2D as compared to healthy subjects. The minimum concentrations were reached at a later time point (4 vs. 2 h in healthy subjects) and the plasma increase at late time points is reduced, suggesting a diminished β-oxidation. Substrate oxidation measures showed that T2D have a significant lower RQ, indicating that T2D use a higher fat% and a lower carbohydrate% as an energy source compared to healthy subjects. The response to PFT revealed that ALAT and ASAT had a higher amplitude to PFT in T2D as compared to healthy subjects. Together these data further confirmed a reduced *hepatic tissue injury control* in T2D.

#### Systemic stress

Finally, in response to PFT, several markers related to *oxidative stress* showed a differential response in T2D as compared to healthy subjects. Glutathione ratio and levels of ribose were significantly lower in T2D as compared to healthy subjects. The response of erythronic acid was found to be strikingly different between the two groups. Healthy subjects showed a clear response (cluster 4) to PFT, whereas this response was lacking in T2D. Finally, CRP levels, a biomarker representing the process of *inflammation,* were significantly higher upon PFT in T2D as compared to healthy subjects.

### Comparison of glucose and insulin PFT responses to OGTT

**Fig. 5:**
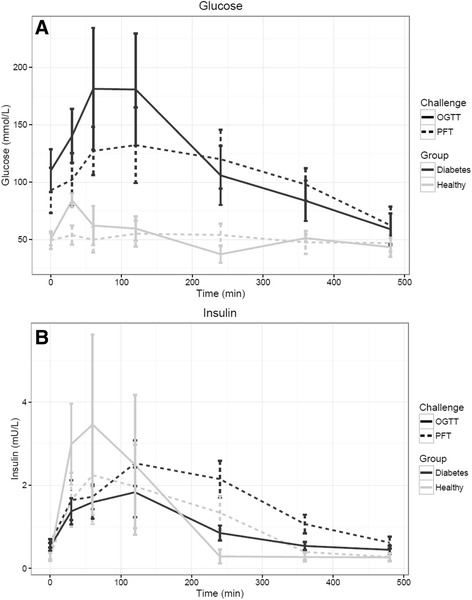
**a** Average glucose response in OGTT vs PhenFlex test in 20 diabetic type 2 male patients and 20 healthy male volunteers. **b** Average insulin response in OGTT vs. PhenFlex test in 20 diabetic type 2 male patients and 20 healthy male volunteers. Red = average diabetes type 2 response. Blue = average healthy response. Solid line = PhenFlex test. Broken line = OGTT

Similar glucose and insulin patterns were observed between the two subjects groups (Fig. 5a, b). Although, plasma glucose and insulin concentrations in T2D and plasma glucose in healthy subjects were higher in response to OGTT than in PFT. Indexes related to glucose metabolism that can be calculated from an OGTT were also calculated for PFT and compared (Table 3). DI, ISI, IGI, and MISI were all significantly different between healthy and T2D after both OGTT and PFT. Although the direction of change was the same for all indexes after OGTT and PFT, the absolute values for the indexes were different after the two challenges, especially for DI (6.31 for OGTT vs. 24.63 for PFT in healthy subjects). Furthermore, it was observed that healthy subjects had a remarkable higher standard deviation for the different indexes as compared to T2D.

**Table 3:** Overview of indexes related to insulin resistance, insulin secretion, and β cell function and their values in response to OGTT as compared to PFT that both contain 75 g of glucose in 20 healthy male subjects and 20 type 2 diabetic male subjects

Index	Healthy		T2D		FDR *p* values	
	OGTT	PhenFlex	OGTT	PhenFlex	OGTT	PhenFlex
DI	6.31 ± 3.4	24.63 ± 41.7	0.51 ± 0.2	1.55 ± 0.8	0	0
IGI	1.28 ± 0.6	3.83 ± 5.7	0.26 ± 0.1	0.91 ± 0.5	0	0.00005
Matsuda	5.83 ± 3.4	7.40 ± 6.0	2.13 ± 1.0	1.84 ± 0.7	0.00002	0
MISI	−1.65 ± 1.1	−1.13 ± 1.2	−0.58 ± 1.2	−0.14 ± 0.3	0	0.00006

Data are presented as mean ± SD. The column FDR *p* values shows the FDR corrected *p*-values after statistical evaluation of indexes in healthy vs T2D per index and per challenge test

*DI* disposition index, *IGI* insulinogenic index, *MISI* muscle insulin sensitivity index

## Conclusions

The goal of this clinical study was to characterize PFT by assessing the healthy adaptive capacity of relevant metabolic processes as determined from the literature review [3]. In other words, this study examined to what extent a series of biomarkers, representing the selected health related processes, are responsive to the PhenFlex challenge in our healthy volunteers.

Processes represented in Fig. 2 were found to be modulated by PFT during the 8 h time course except for chronic low-grade inflammation as measured by CRP. These findings, including a lack of response of CRP, are in accordance with what is described in the literature [3]. Importantly, processes of endothelial integrity, represented by sVCAM-1, sICAM-1, SAA, total cholesterol, HDL, and LDL, showed a significant time effect in healthy male volunteers as well as markers related to hepatic tissue injuries such as GGT, ALP, ASAT, and ALAT. Also, most markers related to oxidative stress represented by uric acid, vitamin E, ribose, erythronic acid, and mannose showed a significant time response as well as metabolic flexibility (RQ). These processes are known to be augmented by a mixed meal challenge as compared to single macronutrient challenges such as OGTT and oral lipid tolerance test (OLTT) [3, 8]. Therefore, we conclude that PFT modulated phenotypic flexibility as expected in healthy individuals.

Five distinct response type profiles were identified (Fig. 3) to PFT, which was a comparable number of the six distinct postprandial time course profiles reported by Pellis et al. [7] in response to a mixed meal challenge. However, some differences can be observed between the biomarker responses in the two studies. For example, cluster 5 is a collection of markers such as glucose, insulin and most amino acids that are being represented into two distinct clusters in the Pellis study [7] that differ in the time that minimum values are being reached (4 and 6 h after postprandial challenge). The studies differ in the composition of the challenge tests and study set-up (4 h of fasting after a standardized breakfast vs. overnight fasting). This shows that it is important that a standardized nutritional challenge protocol is being developed in order to compare results between studies and for the interpretation of the challenge test response profiles.

The secondary objective was to investigate whether PFT and defined new biomarkers are useful to demonstrate reduced phenotypic flexibility in metabolically impaired subjects, in this case, T2D, as compared to healthy subjects. The phenotypic flexibility markers showed a more sensitive response than the fasting markers (Fig. 4, Additional file 1: Table S1 and Table 2), shown by 58 significantly different post-challenge time courses upon PFT as compared to 18 parameters that were significantly different at fasting. Based on challenging conditions, our results evidently confirmed that T2D is a systems disease with an impaired adaptive response of pancreas, liver, muscle, adipose, gut, systemic stress, vasculature and kidney (Fig. 4). This is in agreement with the acknowledgement of eight organs that are important players in the hyperglycemic phenotype of T2D [29]. With the exception of brain insulin resistance, all other seven players that have an important role in this hyperglycemic phenotype were identified by looking at the phenotypic flexibility response. Besides the well-known interplay of muscle, liver and β cell, we were able to identify involvement of the fat cell (disturbed lipolysis), gastrointestinal tract (incretin deficiency/resistance), α cell (hyperglucagonemia), and kidney (increased glucose reabsorption) [29]. Subsequently, we also identified “vasculature” as an important player in the phenotype of T2D, which is a widely accepted phenomenon [30]. Furthermore, many of the metabolites that were found to be significantly different between T2D and healthy that were observed in our study are quite well known from metabolic profiling studies in diabetes [31]. Amino acids, in particular, the branched-chain amino acids and glycine, carbohydrates including 1,5-anhydroglucitol and ketone bodies including alpha-hydroxybutyrate all have been identified as (predictive) biomarkers for T2D [31]. Some of the observations, especially related to the gut such as incretins and amino acids absorption may be the consequence of delayed gastric emptying, which is occurring in 30–50% of T2D [32]. A study limitation was that our T2D subjects were on average 16 years older as compared to the healthy subjects and therefore ‘aging’ may partly explain our observations. So far, the effect of aging on phenotypic flexibility has not been studied, so it has to be speculated to what degree this influenced our results. Probably aging deteriorates most health-related processes to a certain extent. Still, our study provides an accurate picture of metabolic dysregulation in diabetes pathology as we know it from literature, indicating the power and sensitivity of the phenotypic flexibility approach.

PFT was compared to OGTT, and although the amplitude and peak of both glucose as well as insulin was reduced in PFT, different insulin sensitivity indexes disclosed similar statistical differences. Based on these results we conclude that PFT can be used to study differences in glucose and insulin metabolism. The reduced glucose and insulin amplitude and delayed peak in response to PFT may be the result of delayed gastric emptying. Furthermore, healthy subjects had a remarkable higher heterogeneity for the different insulin sensitivity indexes both based on OGTT as well as on PhenFlex challenge. This was also checked for the other parameters quantified in this study, but not such differences were being observed for other markers between the two groups. When obvious differences in standard deviation were present, it was either in the healthy group of volunteers as well as in T2D. The current results may suggest that OGTT is more sensitive in the identification of insulin-related effects. The added value of PFT is that it augments processes of endothelial integrity, hepatic tissue injury, oxidative stress as well as metabolic flexibility which are important processes in the determination of metabolic health.

Taken together, PFT thus provides more accurate information on a broad spectrum of phenotypic flexibility processes. We, therefore, propose PFT as a standardized mixed meal tolerance test in metabolic and nutritional studies. The results suggest that PFT is applicable for examining health effects of a broad range of diets and dietary-ingredients and provides valuable additional information as compared to the oral glucose tolerance test.

## Additional files

Additional file 1: Table S1. Health biomarkers related to phenotypic flexibility. (DOCX 42 kb)

Additional file 2: Figure S1 A–F. Graphical overview of statistical outcomes of the response to the PhenFlex challenge of all parameters quantified. (DOCX 500 kb)

## Abbreviations

ALAT: Alanine-aminotransferase
ALP: Alkaline phosphatase
ASAT: Aspartate-aminotransferase
BMI: Body mass index
CHDR: Centre of Human Drug Research
CRP: C-reactive protein
ER: Endoplasmic reticulum
FDR: False discovery rate
FFA: Free fatty acids
GCMS: Gas chromatography–mass spectrometry
GGT: Gamma-glutamyltransferase
GIP: Glucose-dependent insulinotropic polypeptide
GLP1: Glucagon-like peptide-1
HbA1c: Hemoglobin A1c
HOMA-B: Homeostasis Model Assessment of beta cell function
HOMA-IR: Homeostasis Model Assessment of insulin resistance
IGI: Insulinogenic index
ISI: Matsuda index/Insulin sensitivity index
LUMC: Leiden University Medical Centre
MISI: Muscle Insulin Sensitivity Index
MSD: Meso scale discovery
MUFA: Monounsaturated fatty acids
NEFA: Non-esterified fatty acids
OGTT: Oral glucose tolerance test
PFT: PhenFlex test or standardized mixed meal tolerance test
RQ: Respiration quotient
SAA: Serum amyloid A
SFA: Saturated fatty acids
sICAM: Secreted intercellular adhesion molecule-1
sVCAM: Secreted vascular adhesion molecule-1
T2D: Type 2 diabetes/type 2 diabetic(s)
TG: Triglycerides

## References

[1] Huber M, Knottnerus JA, Green L, van der Horst H, Jadad AR, Kromhout D, et al. How should we define health? BMJ [Internet]. 2011 [cited 2016 May 4];343:d4163. Available from: http://www.ncbi.nlm.nih.gov/pubmed/21791490 10.1136/bmj.d4163 21791490

[2] van Ommen B, Keijer J, Heil SG, Kaput J. Challenging homeostasis to define biomarkers for nutrition related health. Mol. Nutr. Food Res. [Internet]. 2009 [cited 2016 May 4];53:795–804. Available from: http://www.ncbi.nlm.nih.gov/pubmed/19517455 10.1002/mnfr.200800390 19517455

[3] Stroeve JHM, van Wietmarschen H, Kremer BHA, van Ommen B, Wopereis S. Phenotypic flexibility as a measure of health: the optimal nutritional stress response test. Genes Nutr. [Internet]. 2015 [cited 2016 May 4];10:459. Available from: http://www.ncbi.nlm.nih.gov/pubmed/25896408 10.1007/s12263-015-0459-1 PMC4404421 25896408

[4] van Ommen B, van der Greef J, Ordovas JM, Daniel H. Phenotypic flexibility as key factor in the human nutrition and health relationship. Genes Nutr. [Internet]. 2014 [cited 2016 May 4];9:423. Available from: http://www.ncbi.nlm.nih.gov/pubmed/25106484 10.1007/s12263-014-0423-5 PMC4172643 25106484

[5] van Ommen B, Wopereis S. Next-generation biomarkers of health. Nestlé Nutr. Inst. Work. Ser. [Internet]. 2016 [cited 2016 May 10];84:25–33. Available from: https://www.ncbi.nlm.nih.gov/pubmed/26764470 10.1159/000436949 26764470

[6] Kardinaal AFM, van Erk MJ, Dutman AE, Stroeve JHM, van de Steeg E, Bijlsma S, et al. Quantifying phenotypic flexibility as the response to a high-fat challenge test in different states of metabolic health. FASEB J. [Internet]. 2015 [cited 2016 May 4];29:4600–13. Available from: http://www.ncbi.nlm.nih.gov/pubmed/26198450 10.1096/fj.14-269852 26198450

[7] Pellis L, van Erk MJ, van Ommen B, Bakker GCM, Hendriks HFJ, Cnubben NHP, et al. Plasma metabolomics and proteomics profiling after a postprandial challenge reveal subtle diet effects on human metabolic status. Metabolomics [Internet]. 2012 [cited 2016 May 4];8:347–59. Available from: http://www.ncbi.nlm.nih.gov/pubmed/22448156 10.1007/s11306-011-0320-5 PMC3291817 22448156

[8] Wopereis S, Wolvers D, van Erk M, Gribnau M, Kremer B, van Dorsten FA, et al. Assessment of inflammatory resilience in healthy subjects using dietary lipid and glucose challenges. BMC Med. Genomics [Internet]. 2013 [cited 2016 May 10];6:44. Available from: http://www.ncbi.nlm.nih.gov/pubmed/24160467 10.1186/1755-8794-6-44 PMC4015956 24160467

[9] Krug S, Kastenmüller G, Stückler F, Rist MJ, Skurk T, Sailer M, et al. The dynamic range of the human metabolome revealed by challenges. FASEB J. [Internet]. 2012 [cited 2016 May 10];26:2607–19. Available from: http://www.ncbi.nlm.nih.gov/pubmed/22426117 10.1096/fj.11-198093 22426117

[10] Esser D, van Dijk SJ, Oosterink E, Lopez S, Müller M, Afman LA. High fat challenges with different fatty acids affect distinct atherogenic gene expression pathways in immune cells from lean and obese subjects. Mol. Nutr. Food Res. [Internet]. 2015 [cited 2016 May 10];59:1563–72. Available from: http://www.ncbi.nlm.nih.gov/pubmed/25913848 10.1002/mnfr.201400853 25913848

[11] Morris C, O’Grada C, Ryan M, Roche HM, Gibney MJ, Gibney ER, et al. Identification of differential responses to an oral glucose tolerance test in healthy adults. PLoS One [Internet]. 2013 [cited 2016 May 10];8:e72890. Available from: http://www.ncbi.nlm.nih.gov/pubmed/23991163 10.1371/journal.pone.0072890 PMC3749984 23991163

[12] Rhee EP, Cheng S, Larson MG, Walford GA, Lewis GD, McCabe E, et al. Lipid profiling identifies a triacylglycerol signature of insulin resistance and improves diabetes prediction in humans. J. Clin. Invest. [Internet]. 2011 [cited 2016 May 4];121:1402–11. Available from: http://www.ncbi.nlm.nih.gov/pubmed/21403394 10.1172/JCI44442 PMC3069773 21403394

[13] Shaham O, Wei R, Wang TJ, Ricciardi C, Lewis GD, Vasan RS, et al. Metabolic profiling of the human response to a glucose challenge reveals distinct axes of insulin sensitivity. Mol. Syst. Biol. [Internet]. 2008 [cited 2016 May 10];4:214. Available from: http://www.ncbi.nlm.nih.gov/pubmed/18682704 10.1038/msb.2008.50 PMC2538910 18682704

[14] Bondia-Pons I, Nordlund E, Mattila I, Katina K, Aura A-M, Kolehmainen M, et al. Postprandial differences in the plasma metabolome of healthy Finnish subjects after intake of a sourdough fermented endosperm rye bread versus white wheat bread. Nutr. J. [Internet]. 2011 [cited 2016 May 10];10:116. Available from: http://www.ncbi.nlm.nih.gov/pubmed/22011443 10.1186/1475-2891-10-116 PMC3214176 22011443

[15] Cruz-Teno C, Pérez-Martínez P, Delgado-Lista J, Yubero-Serrano EM, García-Ríos A, Marín C, et al. Dietary fat modifies the postprandial inflammatory state in subjects with metabolic syndrome: the LIPGENE study. Mol. Nutr. Food Res. [Internet]. 2012 [cited 2016 May 10];56:854–65. Available from: http://www.ncbi.nlm.nih.gov/pubmed/22707261 10.1002/mnfr.201200096 22707261

[16] Esser D, Mars M, Oosterink E, Stalmach A, Müller M, Afman LA. Dark chocolate consumption improves leukocyte adhesion factors and vascular function in overweight men. FASEB J. [Internet]. 2014 [cited 2016 May 10];28:1464–73. Available from: http://www.ncbi.nlm.nih.gov/pubmed/24302679 10.1096/fj.13-239384 24302679

[17] Blanco-Rojo R, Alcala-Diaz JF, Wopereis S, Perez-Martinez P, Quintana-Navarro GM, Marin C, et al. The insulin resistance phenotype (muscle or liver) interacts with the type of diet to determine changes in disposition index after 2 years of intervention: the CORDIOPREV-DIAB randomised clinical trial. Diabetologia [Internet]. 2016 [cited 2016 May 10];59:67–76. Available from: http://www.ncbi.nlm.nih.gov/pubmed/26474775 10.1007/s00125-015-3776-4 26474775

[18] Huber M, Bakker MH, Dijk W, Prins HAB, Wiegant FAC. The challenge of evaluating health effects of organic food; operationalisation of a dynamic concept of health. J. Sci. Food Agric. [Internet]. 2012 [cited 2016 May 10];92:2766–73. Available from: http://www.ncbi.nlm.nih.gov/pubmed/22252459 10.1002/jsfa.5563 22252459

[19] Rasmussen MH, Ho KK, Kjems L, Hilsted J. Serum growth hormone-binding protein in obesity: effect of a short-term, very low calorie diet and diet-induced weight loss. J. Clin. Endocrinol. Metab. [Internet]. 1996 [cited 2016 Dec 19];81:1519–24. Available from: http://www.ncbi.nlm.nih.gov/pubmed/8636361. 10.1210/jcem.81.4.8636361 8636361

[20] Thomsen C, Rasmussen O, Christiansen C, Pedersen E, Vesterlund M, Storm H, et al. Comparison of the effects of a monounsaturated fat diet and a high carbohydrate diet on cardiovascular risk factors in first degree relatives to type-2 diabetic subjects. Eur. J. Clin. Nutr. [Internet]. 1999 [cited 2016 Dec 19];53:818–23. Available from: http://www.ncbi.nlm.nih.gov/pubmed/10556990 10.1038/sj.ejcn.1600855 10556990

[21] Esposito K, Nappo F, Giugliano F, Di Palo C, Ciotola M, Barbieri M, et al. Meal modulation of circulating interleukin 18 and adiponectin concentrations in healthy subjects and in patients with type 2 diabetes mellitus. Am. J. Clin. Nutr. [Internet]. 2003 [cited 2016 Dec 19];78:1135–40. Available from: http://www.ncbi.nlm.nih.gov/pubmed/14668275 10.1093/ajcn/78.6.1135 14668275

[22] Blond E, Maitrepierre C, Normand S, Sothier M, Roth H, Goudable J, et al. A new indirect calorimeter is accurate and reliable for measuring basal energy expenditure, thermic effect of food and substrate oxidation in obese and healthy subjects. E. Spen. Eur. E. J. Clin. Nutr. Metab. [Internet]. Elsevier; 2011 [cited 2016 May 10];6:e7–15. Available from: http://linkinghub.elsevier.com/retrieve/pii/S175149911000065X

[23] Matsuda M, DeFronzo RA. Insulin sensitivity indices obtained from oral glucose tolerance testing: comparison with the euglycemic insulin clamp. Diabetes Care [Internet]. 1999 [cited 2016 May 10];22:1462–70. Available from: http://www.ncbi.nlm.nih.gov/pubmed/10480510 10.2337/diacare.22.9.1462 10480510

[24] Abdul-Ghani MA, Matsuda M, Balas B, DeFronzo RA. Muscle and liver insulin resistance indexes derived from the oral glucose tolerance test. Diabetes Care [Internet]. 2007 [cited 2016 May 10];30:89–94. Available from: http://www.ncbi.nlm.nih.gov/pubmed/17192339 10.2337/dc06-1519 17192339

[25] DeFronzo RA, Banerji MA, Bray GA, Buchanan TA, Clement S, Henry RR, et al. Determinants of glucose tolerance in impaired glucose tolerance at baseline in the Actos Now for Prevention of Diabetes (ACT NOW) study. Diabetologia [Internet]. 2010 [cited 2016 Jul 8];53:435–45. Available from: http://www.ncbi.nlm.nih.gov/pubmed/20012012 10.1007/s00125-009-1614-2 20012012

[26] Song Y, Manson JE, Tinker L, Howard B V, Kuller LH, Nathan L, et al. Insulin sensitivity and insulin secretion determined by homeostasis model assessment and risk of diabetes in a multiethnic cohort of women: the Women’s Health Initiative Observational Study. Diabetes Care [Internet]. 2007 [cited 2016 May 10];30:1747–52. Available from: https://www.ncbi.nlm.nih.gov/pubmed/17468352 10.2337/dc07-0358 PMC1952235 17468352

[27] Tang W, Fu Q, Zhang Q, Sun M, Gao Y, Liu X, et al. The association between serum uric acid and residual β-cell function in type 2 diabetes. J. Diabetes Res. [Internet]. 2014 [cited 2016 May 10];2014:709691. Available from: http://www.ncbi.nlm.nih.gov/pubmed/24971368 10.1155/2014/709691 PMC4058242 24971368

[28] Hanson RL, Pratley RE, Bogardus C, Narayan KM, Roumain JM, Imperatore G, et al. Evaluation of simple indices of insulin sensitivity and insulin secretion for use in epidemiologic studies. Am. J. Epidemiol. [Internet]. 2000 [cited 2016 May 10];151:190–8. Available from: http://www.ncbi.nlm.nih.gov/pubmed/10645822 10.1093/oxfordjournals.aje.a010187 10645822

[29] Defronzo RA. Banting Lecture. From the triumvirate to the ominous octet: a new paradigm for the treatment of type 2 diabetes mellitus. Diabetes [Internet]. 2009 [cited 2016 Jul 22];58:773–95. Available from: http://www.ncbi.nlm.nih.gov/pubmed/19336687 10.2337/db09-9028 PMC2661582 19336687

[30] Tousoulis D, Papageorgiou N, Androulakis E, Siasos G, Latsios G, Tentolouris K, et al. Diabetes mellitus-associated vascular impairment: novel circulating biomarkers and therapeutic approaches. J. Am. Coll. Cardiol. [Internet]. 2013 [cited 2016 Oct 21];62:667–76. Available from: https://www.ncbi.nlm.nih.gov/pubmed/23948511 10.1016/j.jacc.2013.03.089 23948511

[31] Suhre K. Metabolic profiling in diabetes. J. Endocrinol. [Internet]. 2014 [cited 2016 Jul 22];221:R75–85. Available from: http://www.ncbi.nlm.nih.gov/pubmed/24868111 10.1530/JOE-14-0024 24868111

[32] Marathe CS, Rayner CK, Jones KL, Horowitz M. Relationships between gastric emptying, postprandial glycemia, and incretin hormones. Diabetes Care [Internet]. 2013 [cited 2016 Oct 4];36:1396–405. Available from: http://www.ncbi.nlm.nih.gov/pubmed/23613599 10.2337/dc12-1609 PMC3631884 23613599

