# Supplementary material for: Multi-parameter comparison of a standardized mixed meal tolerance test in healthy and type 2 diabetic subjects: the PhenFlex challenge
Source: Genes Nutr. 2017 Aug 29;12:21. doi: 10.1186/s12263-017-0570-6 (PMC5576306; doi:10.1186/s12263-017-0570-6)
Supplement: Supplementary file 1 — Health biomarkers related to phenotypic flexibility. (DOCX 42 kb) [file 12263_2017_570_MOESM1_ESM.docx]

**Supplemental Table 1.** Health biomarkers related to phenotypic flexibility markers. Overview of observed statistical responses of parameters related to gut, adipose tissue, systemic stress, muscle, liver, kidney, vasculature and pancreas in healthy subjects in response to PFT challenge and what type of response these parameters show (response cluster). Furthermore, the table indicates whether significantly different PFT challenge test responses were reported (interaction = i) and/or significant differences in postprandial levels were reported (group = g).

| *Organ system* | *Process* | *Plasma Markers* | *Response Cluster*  *H - D* | |
| --- | --- | --- | --- | --- |
| Gut | Host-microbe interaction | Indole-3-propionic acid | 5 |  |
|  | Gut hormone production | GLP-1 | 4 | g^***^ |
|  |  | GIP | 4 |  |
| Adipose | Lipolysis & Lipotoxicity | NEFA | 1 | i^***^ |
| tissue |  | C12:0 fatty acid | 3 | i^***^ |
|  |  | C14:0 fatty acid | 3 | i^***^ |
|  |  | C16:0 fatty acid | 1 | i^***^ |
|  |  | C16:1 fatty acid | 3 | i^***^ |
|  |  | C17:0 fatty acid | 3 | i^***^ |
|  |  | C18:0 fatty acid | 3 | i^***^ |
|  |  | C18:1 fatty acid | 1 | i^***^ |
|  |  | C18:2 fatty acid | 1 | i^***^ |
|  |  | C20:4 fatty acid | 3 |  |
|  |  | C22:6 fatty acid | 3 |  |
|  |  | 1,2 DG-MwND592 | 1 |  |
|  |  | 1,2 DG-MwND618 | 1 |  |
|  |  | 1,2 DG-MwND620 | 1 |  |
|  |  | 1,3 DG-MwND592 | 1 |  |
|  |  | 1,3 DG-MwND620 | 1 |  |
|  |  | 1-Monolineoylglycerol | 1 |  |
|  |  | 1-Monooleoylglycerol | 1 |  |
|  |  | 1-Monopalmitoylglycerol |  |  |
|  |  | 1-Monostearoylglycerol |  |  |
|  |  | 2-Monopalmitoylglycerol |  |  |
|  |  | Glycerol | 1 | i^**^ |
|  | Adipose insulin sensitivity | NEFAxfasting insulin | na |  |
|  | Estimated SCD activity | C16:1 / C16:0 fatty acid | na |  |
|  |  | C18:1 / C18:0 fatty acid | na |  |
|  | Lipokine & Adipokine | Adiponectin |  |  |
|  | production | C16:1n7 | 3 |  |
|  |  | Leptin | 1 | i^***^ |
|  |  | Resistin |  |  |
| Systemic | Systemic insulin | HbA1c | na |  |
| stress | sensitivity | HOMA-IR | na | g^***^ |
|  |  | Glucose | 5 | i^***^ |
|  |  | Fructose | 5 | g^**^ |
|  |  | Matsuda index | na | g^***^ |
|  |  | Fructosamine |  |  |
|  | Oxidative stress | Uric acid | 5 |  |
|  |  | Vitamin E | 3 |  |
|  |  | Glutathione ratio |  | g^*^ |
|  |  | Ribose | 1 | g^*^ |
|  |  | Pseudo uridine |  |  |
|  |  | Hypoxanthine |  |  |
|  |  | Erythronic acid | 4 | i^***^ |
|  |  | Mannose | 3 | i^*^ |
|  | Inflammation | CRP |  | i^**^ |
|  | Secondary messengers | Inositol | 2 |  |
|  |  | Myoinositol | 2 |  |
|  | Adaptation carb/lipid | Fat oxidation | na | g^*^ |
|  |  | Respiratory quotient | na | g^*^ |
|  |  | Carbohydrate oxidation | na | g^*^ |
| Muscle | Protein metabolism: | Isoleucine | 5 | i^***^ |
|  | Branched chain AA | Leucine | 5 | i^***^ |
|  |  | Valine | 5 | i^*^ |
|  |  | 3-Methyl-2-oxovaleric acid | 5 | i^***^ |
|  |  | 4-Methyl-2-oxovaleric acid | 5 | i^***^ |
|  | Protein metabolism: | Alanine | 5 | i^*^ |
|  | Other AA | Asparagine | 5 | i^*^ |
|  |  | Cysteine |  | g^*^ |
|  |  | Glutamine | 2 | i^**^ |
|  |  | Lysine | 5 | i^**^ |
|  |  | Proline | 5 |  |
|  |  | Serine | 5 | i^**^ |
|  |  | Threonine | 5 | i^*^ |
|  |  | Aspartate | 4 |  |
|  |  | Glutamate | 5 | i^**^ |
|  |  | Glycine | 2 | g^*^ |
|  |  | Histidine | 5 |  |
|  |  | Methionine | 5 |  |
|  |  | Phenylalanine | 5 | g^**^ |
|  |  | Tryptophan | 2 |  |
|  |  | Tyrosine | 5 | i^*^ |
|  | Muscle tissue injury | Creatinine | 2 |  |
|  |  | Lactate | 2 |  |
|  |  | 1-Methylhistidine | 2 |  |
|  |  | 3-Methylhistidine | 2 |  |
|  |  | 4-Hydroxyproline | 2 |  |
|  |  | 4-Oxoproline | 3 | g^***^ |
|  |  | Beta-alanine | 3 |  |
|  | Muscle Insulin sensitivity | Muscle IR index | na | g^***^ |
| Liver | Citric acid cycle | Alpha-Ketoglutaric acid | 1 | g^***^ |
|  |  | Citric acid | 3 |  |
|  |  | Succinic acid | 3 | i^***^ |
|  |  | Malate | 3 |  |
|  | Ketogenesis | 3-Hydroxybutanoic acid | 1 | i^***^ |
|  |  | Acetoacetate | 1 | i^***^ |
|  |  | 2-Hydroxybutanoic acid | 1 | i^***^ |
|  | Glycolysis/glyconeogenesis | Pyruvate | 5 | i^**^ |
|  |  | Lactate | 2 |  |
|  |  | Glycerol-3-Phosphate | 1 |  |
|  | Lipoprotein production | Free cholesterol | 1 |  |
|  |  | Spingomyelin (d16:1/16:0) | 1 |  |
|  |  | Spingomyelin (d16:1/18:0) |  |  |
|  |  | Spingomyelin (d16:1/20:0) |  |  |
|  |  | Spingomyelin (d16:1/22:0) |  |  |
|  |  | Spingomyelin (d16:1/24:1) | 1 |  |
|  |  | Spingomyelin (d17:1/16:0) | 1 |  |
|  |  | Spingomyelin (d17:1/24:0) | 1 |  |
|  |  | Spingomyelin (d18:0/16:0) | 1 |  |
|  |  | Spingomyelin (d18:1/16:0) | 1 | g^*^ |
|  |  | Spingomyelin (d18:1/18:0) |  |  |
|  |  | Spingomyelin (d18:1/22:0) | 1 |  |
|  |  | Spingomyelin (d18:1/23:0) | 1 |  |
|  |  | Spingomyelin (d18:1/24:0) | 1 |  |
|  |  | Spingomyelin (d18:2/16:0) | 1 |  |
|  |  | Spingomyelin (d18:2/18:0) |  |  |
|  |  | Spingomyelin (d18:2/20:0) | 1 |  |
|  |  | Spingomyelin (d18:2/24:0) | 1 | g^*^ |
|  |  | TG | 4 |  |
|  | Hepatic tissue injury | GGT | 1 | g^***^ |
|  |  | ALP | 1 |  |
|  |  | ASAT | 1 | i^*^ |
|  |  | ALAT | 1 | g^***^ |
|  | Hepatic insulin sensitivity | Liver IR index | na |  |
| Kidney | (re)Absorption | Creatinine | 2 |  |
|  |  | 1,5-anhydroglucitol |  | g^***^ |
|  | Urea cycle | Aspartic acid | 4 |  |
|  |  | Glutamic acid | 5 |  |
|  |  | Ornitine | 5 | i^***^ |
|  |  | Urea | 2 |  |
|  |  | Albumin | 1 |  |
| Vasculature | Endothelial integrity | SAA | 2 |  |
|  |  | sVCAM-1 | 3 |  |
|  |  | sICAM-1 | 3 | g^**^ |
|  |  | Total cholesterol | 3 |  |
|  |  | HDL | 3 | g^*^ |
|  |  | LDL | 3 | g^*^ |
| Pancreas | Alpha-cell function | Glucagon | 4 | g^*^ |
|  | Beta-cell function | Insulin | 5 | i^***^ |
|  |  | C-peptide | 5 | i^***^ |
|  |  | Disposition index | na | g^***^ |
|  |  | HOMA-B | na | g^**^ |
|  |  | Insulinogenic Index | na | g^***^ |

^*^ Significant differences between fasting values healthy subjects and T2D (p value < 0.05);
^**^ Significant differences between fasting values healthy subjects and T2D (p value < 0.01);
^***^ Significant differences between fasting values healthy subjects and T2D (p value < 0.001)
