# Supplementary material for: Multi-parameter comparison of a standardized mixed meal tolerance test in healthy and type 2 diabetic subjects: the PhenFlex challenge
Source: Genes Nutr. 2017 Aug 29;12:21. doi: 10.1186/s12263-017-0570-6 (PMC5576306; doi:10.1186/s12263-017-0570-6)

**Figure S1:** **Graphical overview of statistical outcomes of the response to the PhenFlex challenge of all 132 quantified parameters.**

Figure S1 (A-G) visualizes the statistical outcomes of the biomarker response to the PhenFlex test. Per measured parameter from left to right the following statistical outcomes are being shown: 1) group effect, healthy vs type 2 diabetes; 2) time effect, by taking the averages of the seven time point combinations and subtracting the overall mean; and 3) the group * time interaction effect by taking the averages of all 14 combinations of health status and time points, and subtracting the overall mean and the effects due to overall differences between healthy and diabetic subjects and the overall differences among the time points. Legend: filled bullets, type 2 diabetic patients; open bullets, healthy subjects; vertical lines, 90% of the between-subject variation; red bullets, statically significant source of variation; black bullets, no statistically source of variation; dashed lines, scaled time effects boundaries for no time effect. When group*time interactions have a significant statistical outcome shown in red, then group effects and time effects are automatically shown in black.
Examples of parameters with significant group effect are CRP and glutathione ratio, examples of parameters with significant time effect are albumin and alkaline phosphatase and examples of parameters with significant group*time interaction effect include C-peptide and non-esterified fatty acids (FFA) (Figure S1A). Figure S1A visualizes statistical outcomes of clinical chemistry and protein parameters; Figure S1B-F visualizes statistical outcomes of metabolites.

Figure S1A


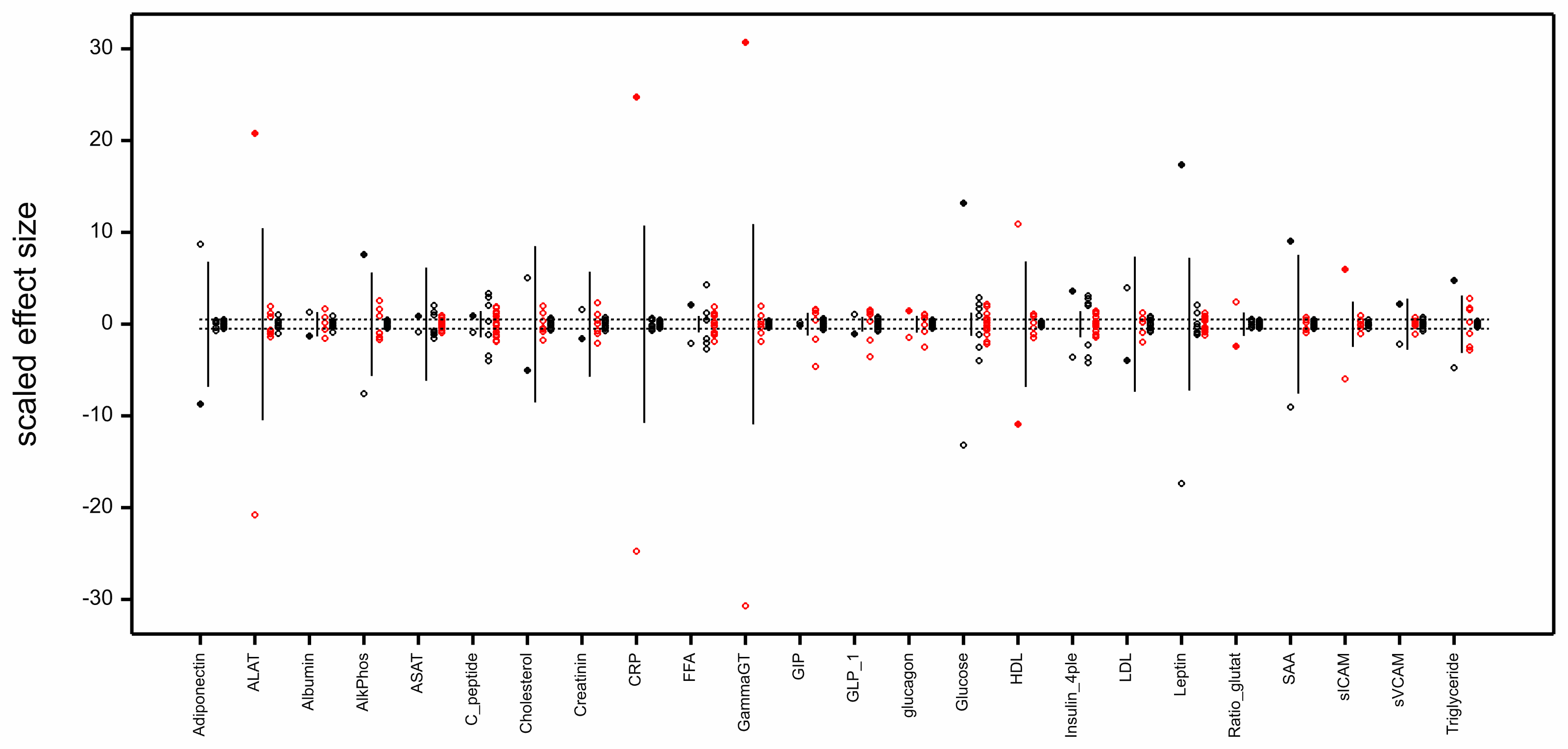


Figure S1B


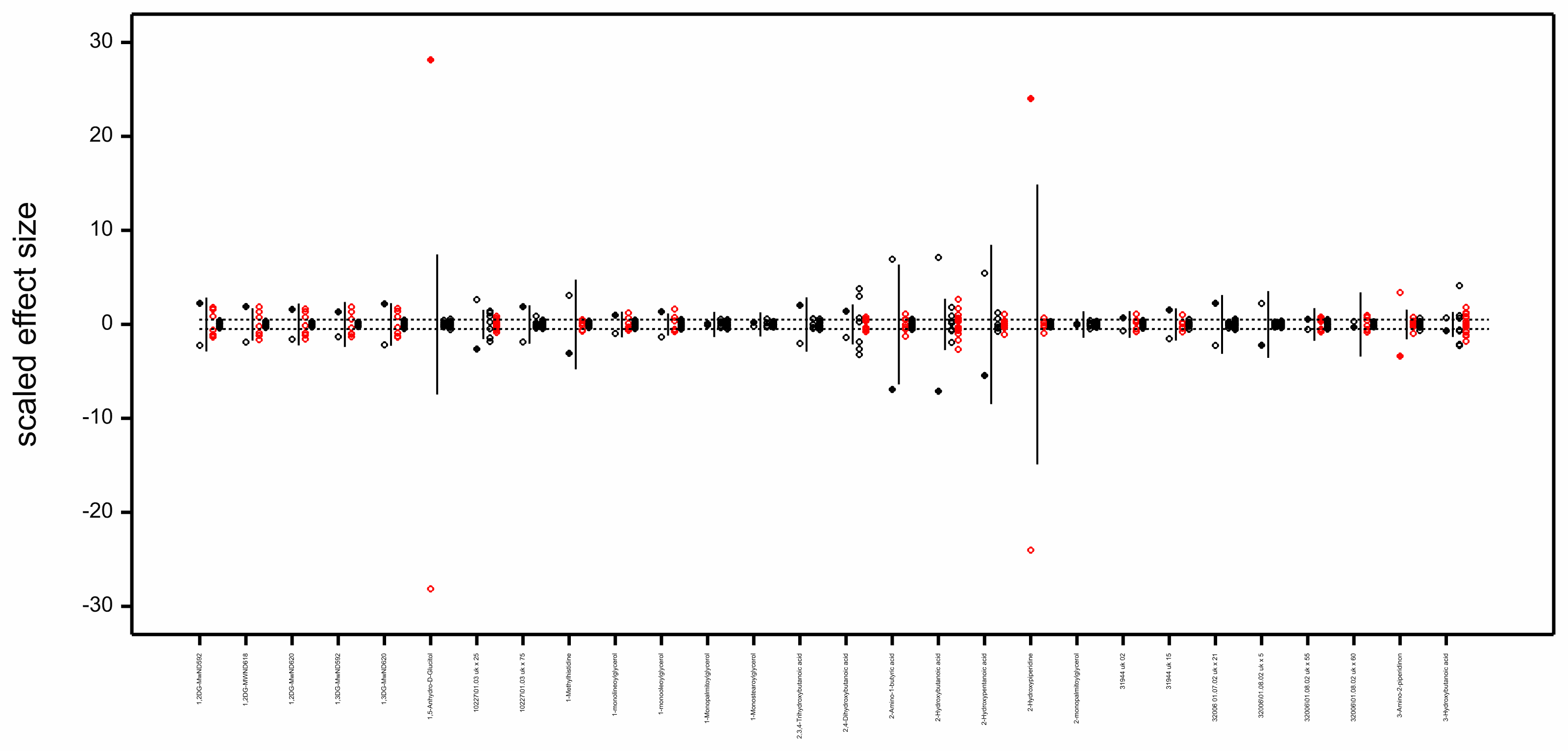


Figure S1C


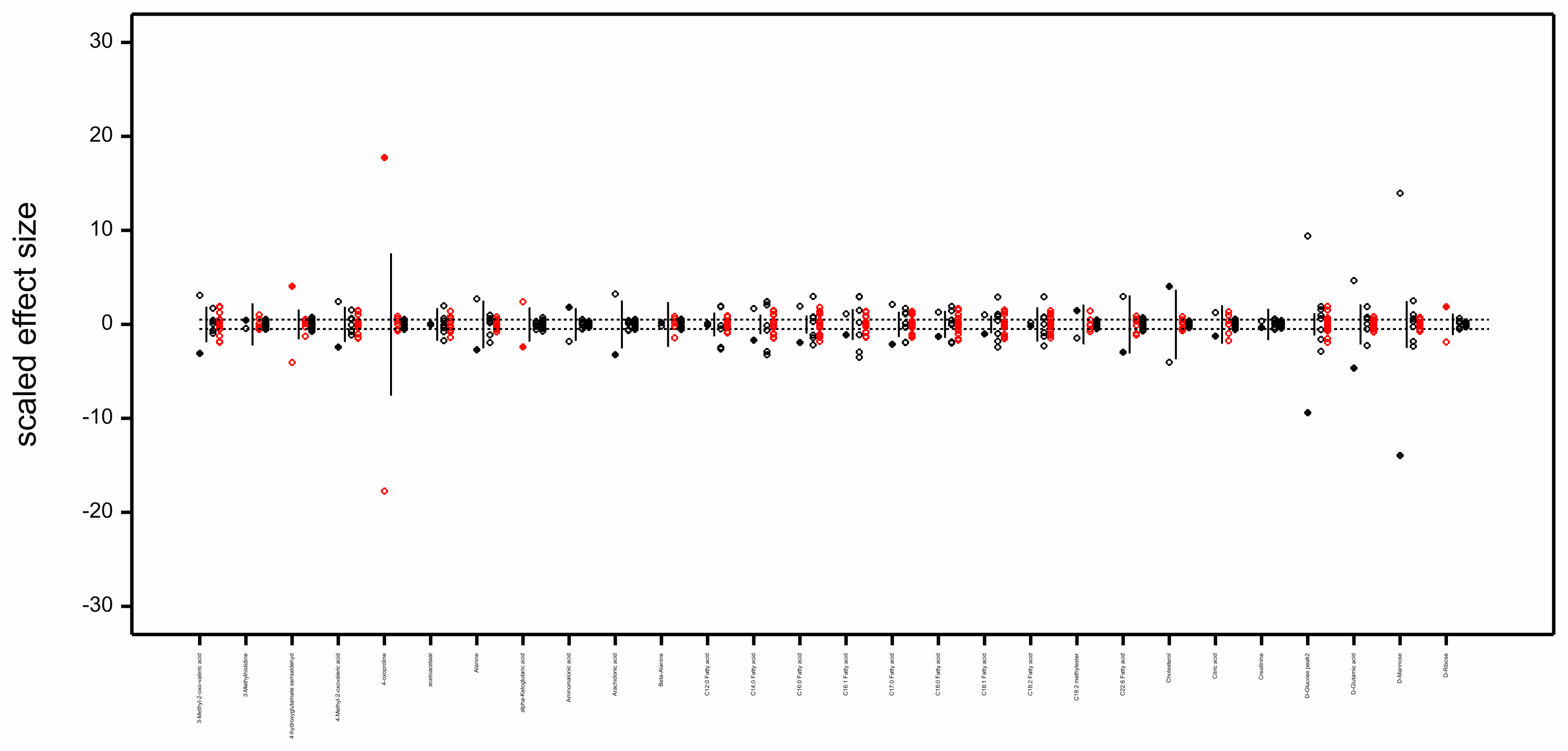


Figure S1D


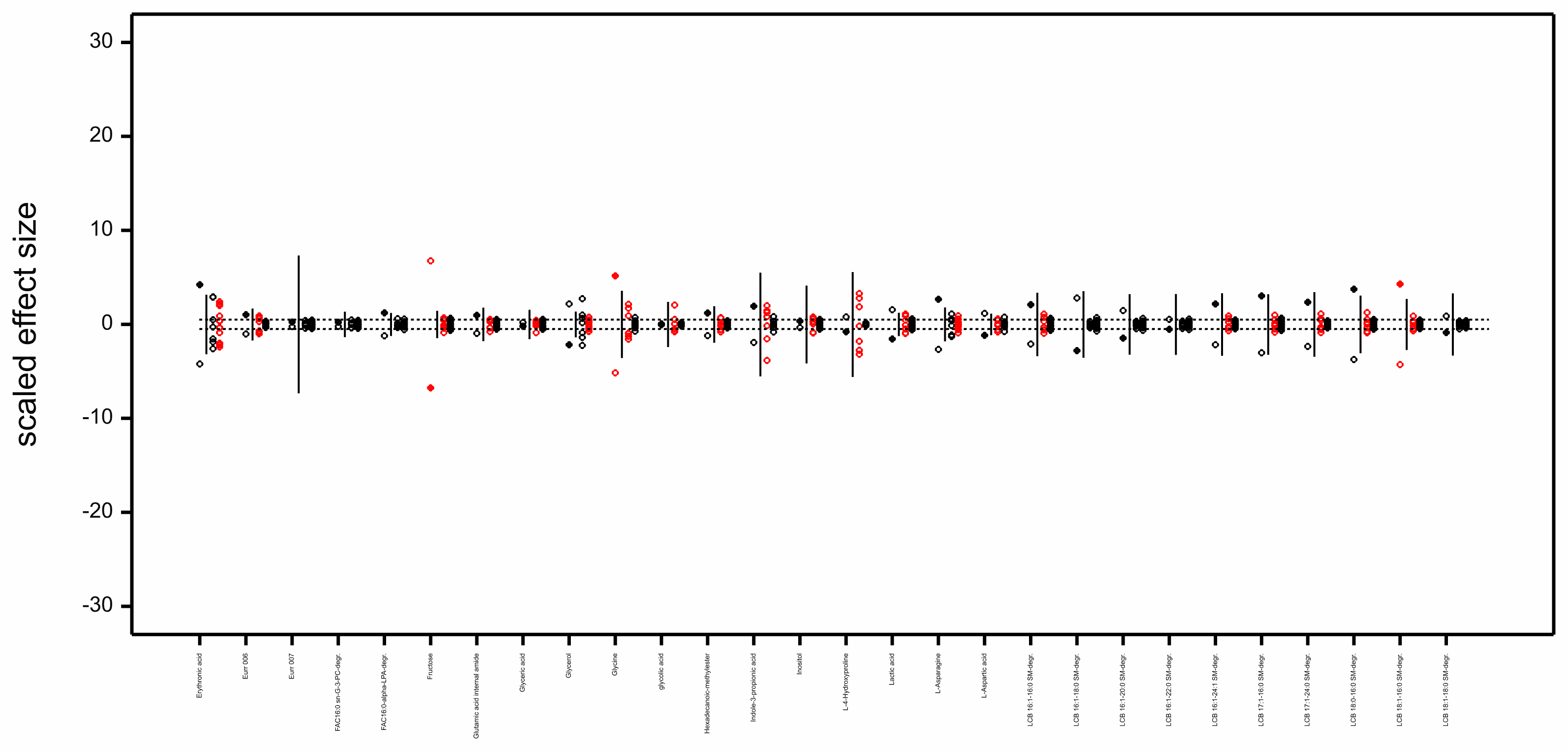


Figure S1E


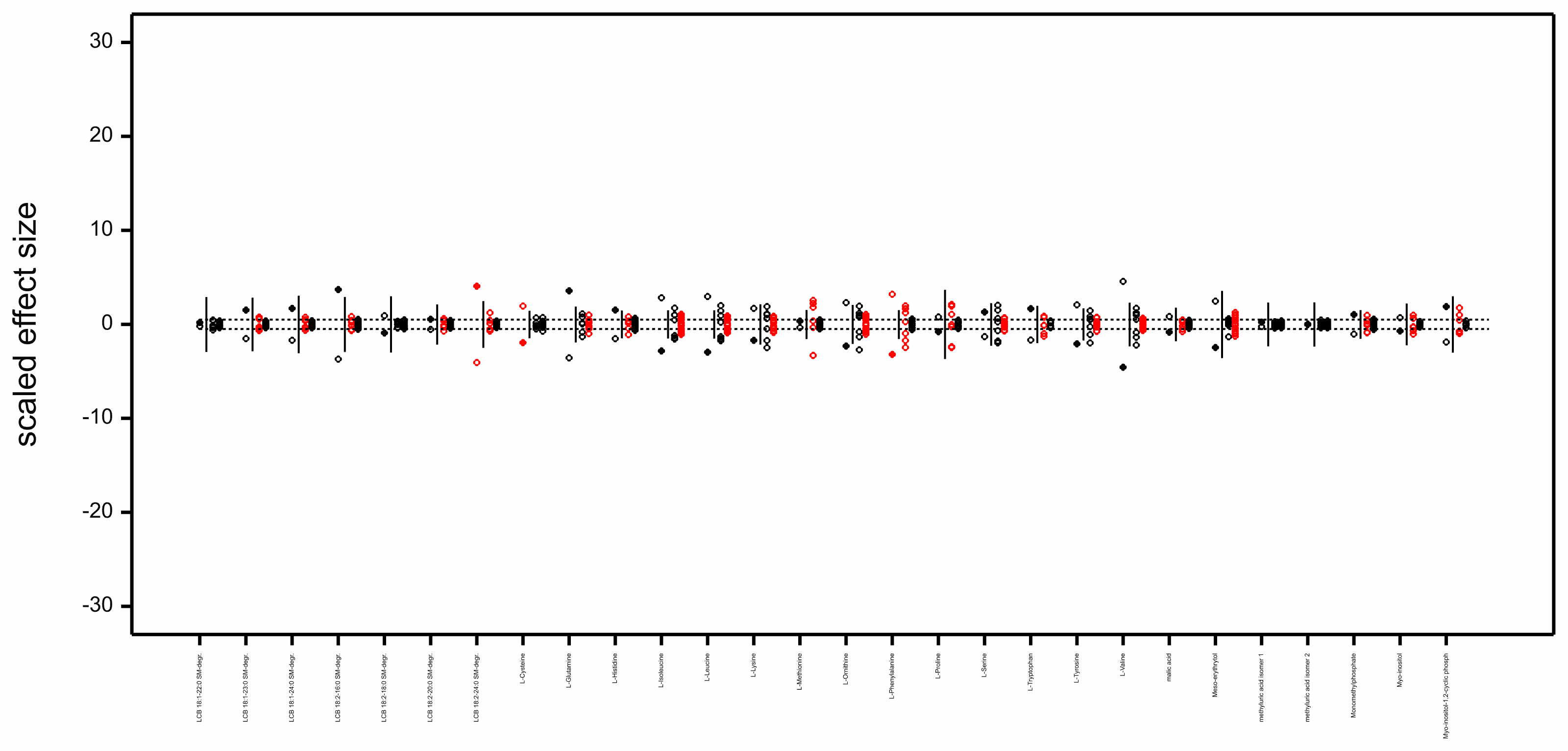


Figure S1F


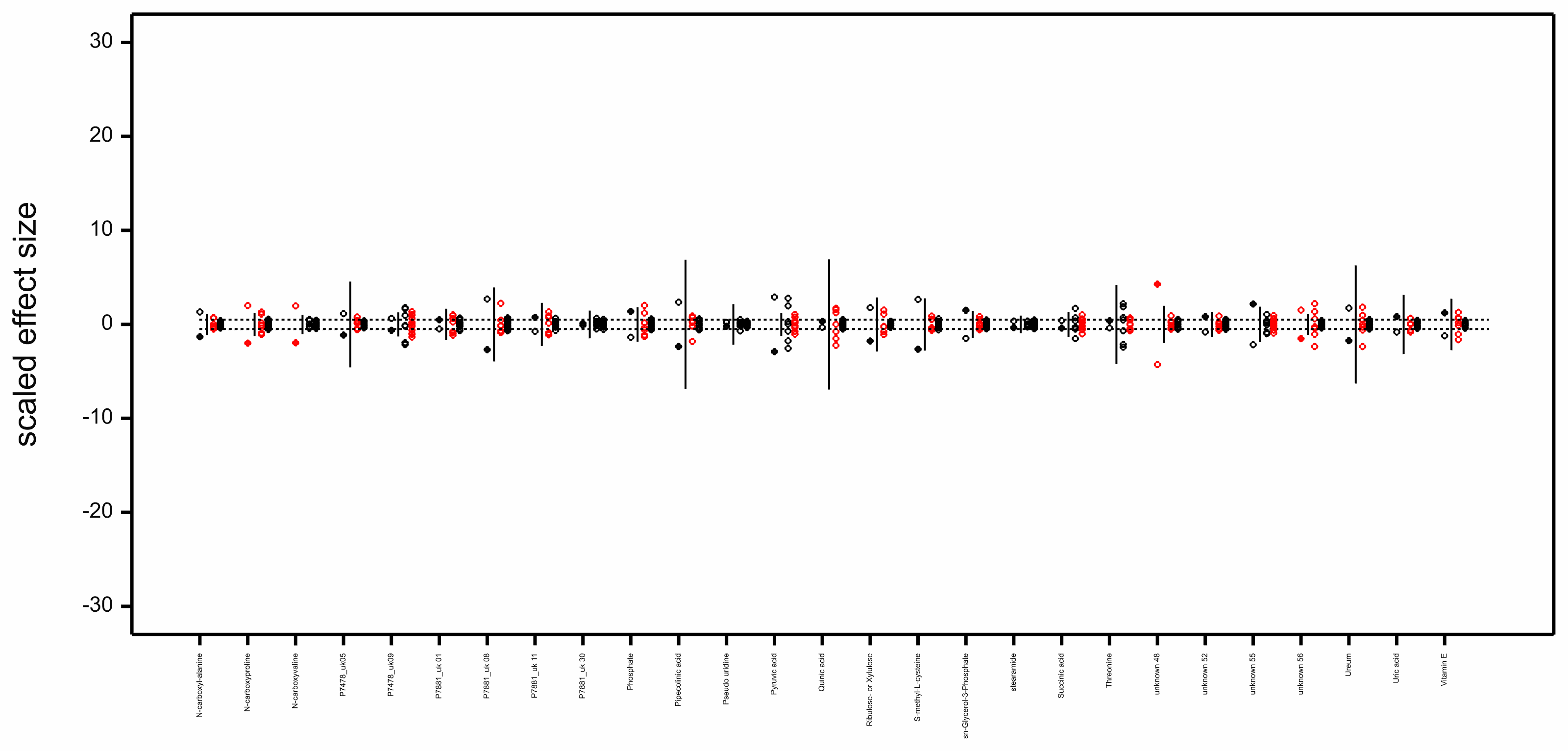

Supplement: Supplementary file 2 — Graphical overview of statistical outcomes of the response to the PhenFlex challenge of all parameters quantified. (DOCX 500 kb) [file 12263_2017_570_MOESM2_ESM.docx]
